# Histopathological and Clinicopathological Correlation of Endoscopic Biopsies of Upper Gastrointestinal Tract Lesions

**DOI:** 10.7759/cureus.108878

**Published:** 2026-05-15

**Authors:** Shivangi Chauhan, Shalini Yadav, Sonal Gupta, Rajni Choudhary, Rajendra Nigam

**Affiliations:** 1 Pathology, Gandhi Medical College, Bhopal, IND; 2 Pathology, Gandhi Medical College and Hamidia Hospital, Bhopal, IND

**Keywords:** adenocarcinoma, clinicopathological correlation, endoscopic biopsy, gastric biopsy, helicobacter pylori, histopathology, neoplastic lesions, squamous cell carcinoma, upper gastrointestinal tract

## Abstract

Background: Upper gastrointestinal (GI) tract disorders are a major cause of morbidity and mortality worldwide. While endoscopic examination provides direct visualization of mucosal lesions, histopathological examination remains the gold standard for definitive diagnosis, particularly for distinguishing between non-neoplastic and neoplastic conditions.

Objective: This study aims to correlate clinical and endoscopic findings with histopathological diagnoses and to assess the prevalence of *Helicobacter pylori* in gastric biopsies.

Materials and methods: A cross-sectional study was conducted at Gandhi Medical College and Associated Hospitals, Bhopal, from May 2023 to October 2024. A total of 100 patients undergoing upper GI endoscopy for various indications were included. Biopsies were taken from lesions in the esophagus, stomach, and duodenum. Tissue samples were routinely processed and stained with hematoxylin and eosin (H&E). Giemsa stain was used to detect *Helicobacter pylori.*

Results: The most common clinical indications for endoscopy were abdominal pain and dyspepsia. Ulcer was the most frequent endoscopic finding (26 (26%)), followed by erosions (14 (14%)). On histopathological evaluation, non-neoplastic lesions predominated in younger age groups (41-50 years), while neoplastic lesions were more frequent in older patients (51-60 years) (p = 0.023). The esophagus was a common site for squamous cell carcinoma, while adenocarcinoma was the most frequent malignancy in the stomach. The diagnostic accuracy of endoscopy for detecting neoplastic lesions, using histopathological diagnosis as the reference standard, was 88%, with a sensitivity of 69%, a specificity of 96%, a positive predictive value (PPV) of 87%, and a negative predictive value (NPV) of 88%. Histopathology detected a higher proportion of neoplastic lesions (29%) compared to endoscopy (23%), highlighting its superior diagnostic accuracy.

Conclusion: Endoscopic biopsy is an essential diagnostic tool that correlates strongly with histopathological findings. While endoscopy is effective for initial screening and identifying suspicious growths, histopathology is crucial for confirming malignancy and identifying specific inflammatory conditions, such as *H. pylori *gastritis. Age and clinical symptoms such as dysphagia are significant predictors of neoplastic pathology in the upper GI tract.

## Introduction

Disorders of the upper gastrointestinal (GI) tract are a significant cause of morbidity and mortality globally, with upper GI cancers, particularly stomach cancer, being among the most prevalent malignancies worldwide [1]. These conditions often present with a common set of symptoms, such as abdominal pain, dyspepsia, and dysphagia, which can make accurate clinical assessment challenging [2].

The introduction of flexible fiberoptic endoscopy in 1968 revolutionized the field by enabling direct visualization of the mucosal surfaces of the esophagus, stomach, and duodenum. Although endoscopy is an important initial diagnostic tool for identifying inflammatory, pre-neoplastic, and neoplastic lesions, histopathological examination is required for definitive diagnosis. Histopathological examination of endoscopic biopsies remains the 'gold standard' for providing a definitive diagnosis and forms the essential basis for treatment planning [3,4].

Furthermore, the role of *Helicobacter pylori* (*H. pylori*) is critical to assess, given its strong link to serious conditions such as peptic ulcers and gastric cancer. Early detection through histopathological correlation enables timely therapeutic intervention and improved patient management [5]. However, limited data exist regarding the clinicopathological correlation of upper GI tract lesions in the Central Indian population, necessitating the present study. The primary objective of the present study was to correlate clinical and endoscopic findings with histopathological diagnoses of upper GI tract lesions. The secondary objective was to assess the prevalence of *H. pylori* in gastric biopsy specimens.

## Materials and methods

Study design and settings

This cross-sectional, hospital-based study was conducted (n=100) in the Department of Pathology in collaboration with the Department of Gastroenterology at Gandhi Medical College and Associated Hospitals, Bhopal. The study duration spanned from May 2023 to October 2024. The study was approved by the Institutional Ethics Committee of Gandhi Medical College, Bhopal (approval number: 18928/MC/IEC/2023 dated 09/05/2023).

Sample size calculation

The sample size was determined using a convenience sampling method, wherein all consecutive patients undergoing upper GI endoscopic biopsy during the study period who fulfilled the inclusion criteria were included in the study.

Inclusion and exclusion criteria

All upper GI endoscopic biopsies of lesions present in the esophagus, stomach, and up to the second part of the duodenum, performed at our center, were included. Inadequate specimens and autolyzed specimens were excluded from the study. Biopsy specimens were considered adequate when sufficient well-preserved mucosal tissue was available for histopathological interpretation.

Procedure and data collection

A detailed clinical history was recorded for each patient. Before the procedure, patients were advised to fast for eight hours. Local anesthesia was administered using Xylocaine gel for the pharynx and hypopharynx. Upper GI endoscopy was performed to visualize the mucosal surface of the esophagus, stomach, and duodenum for abnormalities such as erosions, ulcers, or growth.

Biopsies were obtained using standard biopsy forceps passed through the endoscopic channel. Multiple samples were taken from suspicious lesions, including the central and peripheral portions of ulcers and growths. Typically, 2-4 biopsy fragments were obtained from each suspicious lesion whenever feasible to improve diagnostic yield.

Histopathological analysis

Tissue specimens, typically ranging 1-5 mm in diameter, were immediately fixed for 10% formalin. The samples were processed using an automated tissue processor and embedded in paraffin blocks. Sections of 4-5 µm thickness were cut and stained with hematoxylin and eosin (H&E) for routine histopathological examination [6]. Giemsa staining was used to detect *H. pylori* in gastric biopsies.

Statistical analysis

Data were recorded on a pro forma and managed using Microsoft Excel 2007 (Microsoft Corporation, Redmond, Washington). Statistical analysis included calculating the mean and standard deviation for continuous variables, while descriptive data were presented as frequencies and percentages. IBM SPSS Statistics for Windows, Version 20 (Released 2011; IBM Corp., Armonk, New York) was used for statistical analysis and test of significance. Pictorial representations in the form of bar diagrams and pie charts were provided wherever necessary. A chi-square test or Fisher’s exact test was used for the comparison of categorical variables. A p-value < 0.05 was considered statistically significant.

## Results

The most common age group affected was 51-60 years (60%). Male predominance was observed, with 65% male and 35% female patients (male: female = 1.86:1) (Table 1).

**Table 1:** Demographic profile of the patients

Parameters	Frequency	Percentage
Age groups (years)		
11–20	3	3%
21–30	12	12%
31–40	17	17%
41–50	23	23%
51–60	30	30%
61–70	11	11%
> 70	4	4%
Total	100	100%
Gender		
Male	65	65%
Female	35	35%
Total	100	100%

Among the presenting clinical symptoms, abdominal pain (38%) was the most commonly reported symptom, followed by dysphagia (31%). Multiple symptoms were observed in several patients (Table 2).

**Table 2:** Presenting clinical symptoms among study participants Patients could present with more than one clinical symptom; therefore, cumulative frequencies may exceed the total sample size.

Clinical features	Frequency	Percentage
Abdominal pain	38	38%
Dysphagia	31	31%
Dyspepsia	23	23%
Vomiting	13	13%
Retro-sternal burning	9	9%
Hematemesis	5	5%
Weight loss	6	6%
Diarrhea	6	6%
Loss of appetite	3	3%
Weakness	3	3%
Oral ulcer	1	1%
Total	100	100%

The stomach was the most frequent site for endoscopic biopsies (56%), followed by the esophagus (29%), the duodenum (14%), and the gastroesophageal (GE) junction (1%). Ulcer was the most common upper GI endoscopic finding (26%), followed by erosion (14%). Out of 29 esophageal endoscopic examinations, proliferative growth and ulcero-proliferative growth were the most common findings (each 27.5%), followed by ulcer and erythema (each 10.3%). Out of 56 endoscopic examinations of the stomach, the antrum was the primary site for the biopsy collection, accounting for 83.9% of the total gastric lesion cases. Ulcer was the most common endoscopic finding, occurring in 30.4% of patients, followed by stomach erosion (23.2%). Of 14 duodenal endoscopic examinations, ulcer was the most common finding (42.9%), followed by flattening of the mucosa (21.5%). Only one patient underwent an endoscopic biopsy for the GE junction, and erythema was observed in that case (Tables 3, 4).

**Table 3:** Site-wise endoscopic findings Values are expressed as frequency (percentage). Percentages are calculated column-wise for each anatomical site (esophagus, stomach, duodenum, and GE junction), considering the total number of findings within that site as 100%. Multiple findings may be present in a single patient; therefore, the total number of findings may exceed the number of patients. GE: Gastroesophageal; GI: Gastrointestinal; HILLS: Hill’s classification of the gastroesophageal flap valve.

Upper GI endoscopic findings	Esophagus	Stomach	Duodenum	Gastrointestinal junction	Total
Ulcer	03 (10.3)	17 (30.4)	06 (42.9)	00 (0.0)	26
Erosion	01 (3.5)	13 (23.2)	00 (0.0)	00 (0.0)	14
Erythema	03 (10.3)	05 (8.9)	01 (7.1)	01 (100.0)	10
Ulcero-proliferative growth	08 (27.5)	01 (1.8)	00 (0.0)	00 (0.0)	09
Proliferative growth	08 (27.5)	00 (0.0)	00 (0.0)	00 (0.0)	08
Normal	00 (0.0)	04 (7.1)	01 (7.1)	00 (0.0)	05
Nodular growth	02 (6.9)	03 (5.3)	00 (0.0)	00 (0.0)	05
Diffuse edema	01 (3.5)	03 (5.3)	00 (0.0)	00 (0.0)	04
Diffuse thickening	01 (3.5)	02 (3.6)	00 (0.0)	00 (0.0)	03
Polypoidal growth	01 (3.5)	00 (0.0)	00 (0.0)	00 (0.0)	01
Flattening of the mucosa	00 (0.0)	00 (0.0)	03 (21.5)	00 (0.0)	03
Congestive gastropathy	00 (0.0)	02 (3.6)	00 (0.0)	00 (0.0)	02
HILLS grade 2	00 (0.0)	02 (3.6)	00 (0.0)	00 (0.0)	02
Scalloping	00 (0.0)	00 (0.0)	02 (14.3)	00 (0.0)	02
Stricture	01 (3.5)	00 (0.0)	01 (7.1)	00 (0.0)	02
Diffuse thinning	00 (0.0)	01 (1.8)	00 (0.0)	00 (0.0)	01
Polyp	00 (0.0)	01 (1.8)	00 (0.0)	00 (0.0)	01
Total	29 (100)	56 (100)	14 (100)	01 (100)	100

**Table 4:** Distribution of patients by site of endoscopic gastric biopsies

Gastric endoscopic biopsy sites	Frequency (n=56)	Percentage (%)
Cardia of the stomach	1	1.8%
Fundus of the stomach	3	5.4%
Body of stomach	5	8.9%
Antrum of the stomach	47	83.9%

**Figure 1:**
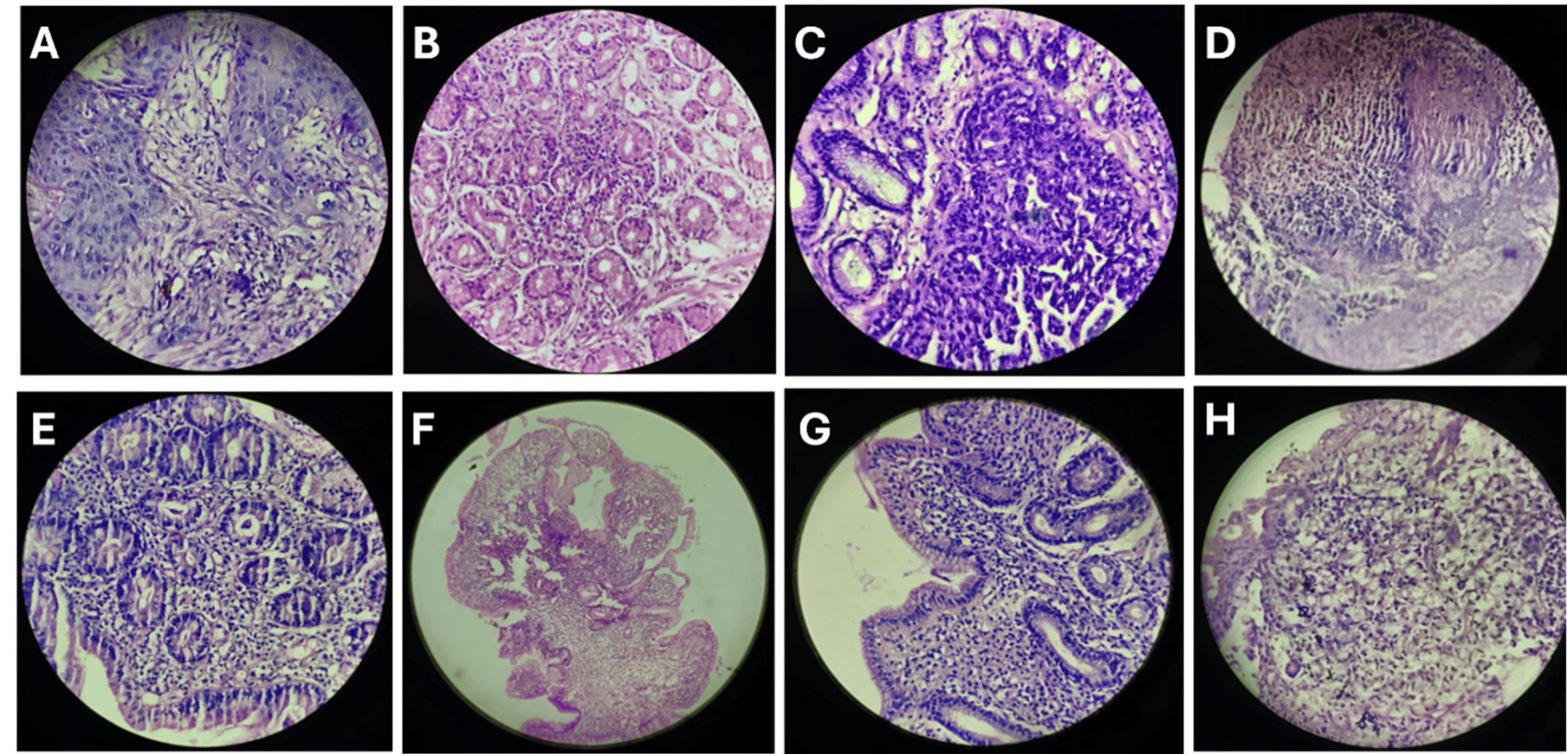
Photomicrographs of different diagnosed cases A: Moderately differentiated squamous cell carcinoma (H&E, 40×) (esophageal biopsy), B: Chronic gastritis (H&E, 40×) (gastric biopsy), C: Adenocarcinoma (H&E, 40×) (gastric biopsy), D: Mucinous adenocarcinoma (H&E, 40×) (gastric biopsy), E: Chronic duodenitis (H&E, 40×), F: Inflammatory gastric polyp (H&E, 10×), G: Celiac disease (H&E, 40×), H: Adenocarcinoma, signet ring type (H&E, 40×). Figures were created using standard microscopy imaging and Microsoft PowerPoint (Microsoft Corporation, Redmond, Washington); no AI-generated images were used.

Overall, on histopathology of upper GI lesions, 71% were diagnosed as non-neoplastic lesions, 4% as pre-neoplastic lesions, and 25% as neoplastic lesions. Overall, "no specific pathology" (32%) was the most common finding under the microscope. In the upper GI, chronic gastritis was the most common diagnosis (13%). The most common non-neoplastic lesion, pre-neoplastic lesion, and neoplastic lesion were nonspecific pathology (32/71), moderate dysplasia (2/4), and moderately differentiated squamous cell carcinoma (SCC) (8/25), respectively (Table 5, Figure 1).

**Table 5:** Histopathological spectrum of upper gastrointestinal lesions Values are expressed as frequency (percentage). Percentages are calculated column-wise for each anatomical site (esophagus, stomach, duodenum, and GE junction), considering the total number of cases at each site as 100%. Multiple histopathological findings may be present in a single patient; therefore, the total number of lesions may exceed the number of patients. GI: Gastrointestinal; GE: Gastroesophageal; SCC: Squamous cell carcinoma; NOS: Not otherwise specified.

Upper GI histopathological diagnosis	Number of cases at each site with percentage				Total
	Esophagus	Stomach	Duodenum	Gastrointestinal junction	
Non-neoplastic lesions	06 (20.7)	50 (89.3)	14 (100)	01 (100.0)	71
No specific pathology	02 (6.9)	25 (44.6)	04 (28.6)	01 (100)	32
Acute esophagitis	03 (10.3)	00 (0.0)	00 (0.0)	00 (0.0)	03
Barrett’s esophagus	01 (3.4)	00 (0.0)	00 (0.0)	00 (0.0)	01
Celiac disease grade 1	00 (0.0)	00 (0.0)	03 (21.5)	00 (0.0)	03
Celiac disease grade 2	00 (0.0)	00 (0.0)	01 (7.1)	00 (0.0)	01
Celiac disease grade 3	00 (0.0)	00 (0.0)	01 (7.1)	00 (0.0)	01
Chronic duodenitis	00 (0.0)	00 (0.0)	05 (35.7)	00 (0.0)	05
Chronic gastritis	00 (0.0)	13 (23.2)	00 (0.0)	00 (0.0)	13
Chronic superficial gastritis	00 (0.0)	10 (17.9)	00 (0.0)	00 (0.0)	10
Hyperplastic polyp	00 (0.0)	01 (1.8)	00 (0.0)	00 (0.0)	01
Inflammatory gastric polyp	00 (0.0)	01 (1.8)	00 (0.0)	00 (0.0)	01
Pre-neoplastic lesions	04 (13.8)	00 (0.0)	00 (0.0)	00 (0.0)	04
Mild dysplasia	01 (3.4)	00 (0.0)	00 (0.0)	00 (0.0)	01
Moderate dysplasia	02 (6.9)	00 (0.0)	00 (0.0)	00 (0.0)	02
Severe dysplasia	01 (3.4)	00 (0.0)	00 (0.0)	00 (0.0)	01
Neoplastic lesions	19 (65.5)	6 (10.7)	00 (0.0)	00 (0.0)	25
Suspicious for malignancy	01 (3.4)	00 (0.0)	00 (0.0)	00 (0.0)	01
Well-differentiated SCC	01 (3.4)	00 (0.0)	00 (0.0)	00 (0.0)	01
Moderately differentiated SCC	08 (27.5)	00 (0.0)	00 (0.0)	00 (0.0)	08
Poorly differentiated SCC	06 (20.7)	00 (0.0)	00 (0.0)	00 (0.0)	06
Well-differentiated adenocarcinoma	01 (3.4)	00 (0.0)	00 (0.0)	00 (0.0)	01
Poorly differentiated adenocarcinoma	01 (3.4)	00 (0.0)	00 (0.0)	00 (0.0)	01
Adenocarcinoma NOS	00 (0.0)	05 (8.9)	00 (0.0)	00 (0.0)	05
Adenocarcinoma signet ring type	01 (3.4)	00 (0.0)	00 (0.0)	00 (0.0)	01
Mucinous adenocarcinoma	00 (0.0)	01 (1.8)	00 (0.0)	00 (0.0)	01
Total	29 (100)	56 (100)	14 (100)	01 (100)	100

Based on the histological diagnosis of 29 esophageal biopsies, we observed 20.7% non-neoplastic, 13.8% pre-neoplastic, and 65.5% neoplastic pathology. Overall, the most common esophageal histopathological diagnosis was moderately differentiated SCC (27.5%). The most common non-neoplastic diagnosis was acute esophagitis (3/6); the pre-neoplastic diagnosis was moderate dysplasia (2/4); and the neoplastic diagnosis was moderately differentiated SCC (8/19) (Table 5, Figure 1).

Based on the histological diagnosis of 56 gastric biopsies, we observed 89.3% non-neoplastic and 10.7% neoplastic pathology. No pre-neoplastic lesion was histopathologically diagnosed. Overall, the most common gastric histopathological diagnosis was of no specific pathology (44.6%), followed by chronic gastritis (23.2%). These are also the most common non-neoplastic diagnoses: no specific pathology (25/50) and neoplastic diagnoses: adenocarcinoma NOS (not otherwise specified) (5/6) (Table 5, Figure 1).

On histological diagnosis of 14 duodenal biopsies, we found all lesions to be non-neoplastic (100%). No pre-neoplastic lesion or neoplastic lesion was histopathologically diagnosed. The most common non-neoplastic diagnosis was chronic duodenitis (35.7%). Only one GE junctional biopsy was performed, which showed non-specific pathology on histopathology (Table 5, Figure 1).

**Figure 2:**
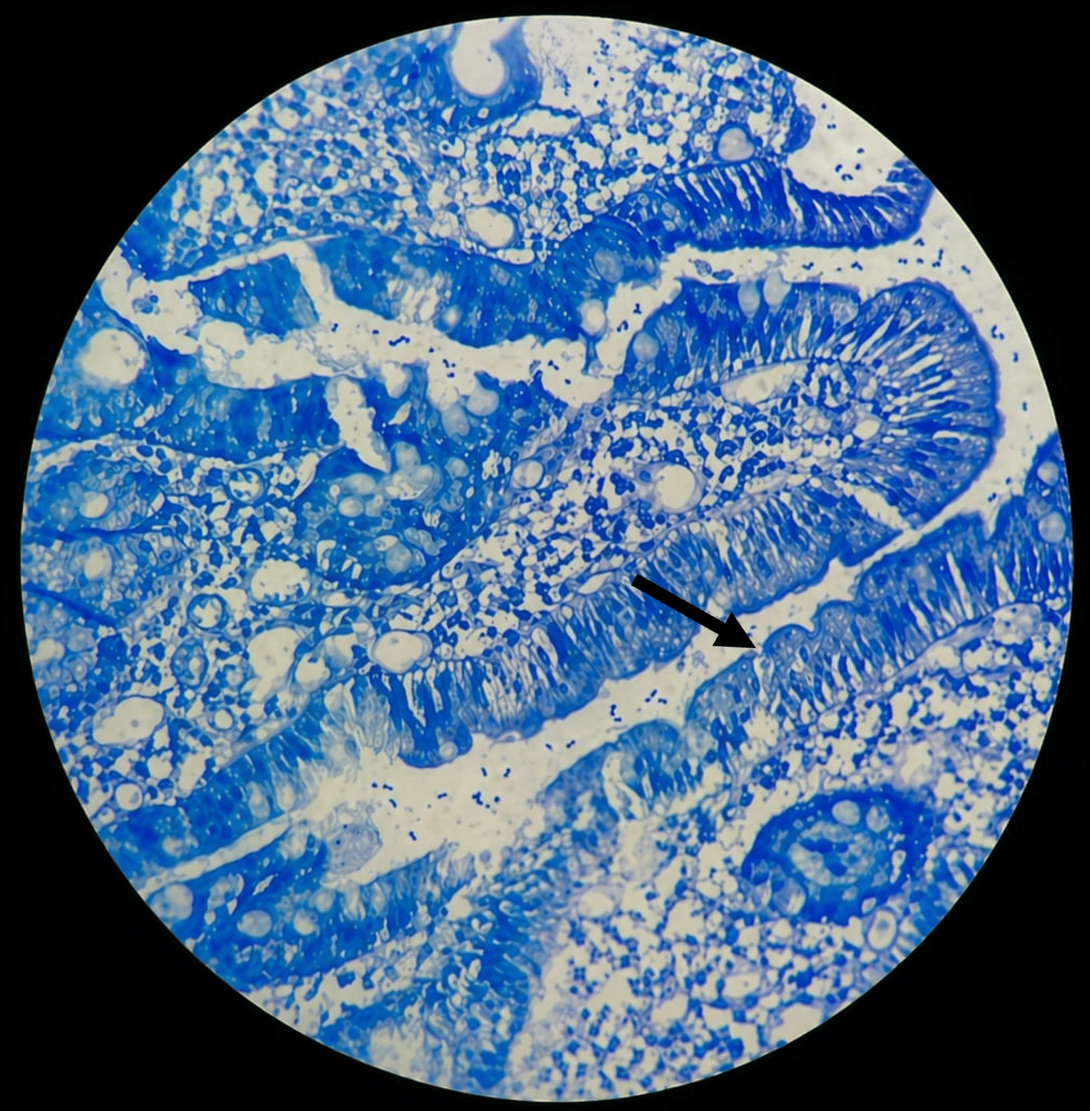
Photomicrograph of H. pylori (black arrow) (Giemsa stain, 100×) Figures were created using standard microscopy imaging and Microsoft PowerPoint (Microsoft Corporation, Redmond, Washington); no AI-generated images were used.

Overall, 7% of cases were *H. pylori* positive. We observed that five *H. pylori*-positive slides belonged to cases of chronic gastritis (i.e., 5/13, 38.46%) and two to cases of chronic superficial gastritis (i.e., 2/10, 20%) (Figure 2).

A total of 86.84% of the patients with abdominal pain, 95.65% of dyspepsia, and 92.30% of vomiting were diagnosed with non-neoplastic upper GI lesions; 87.10% of the patients with dysphagia were diagnosed with pre-neoplastic/neoplastic lesions (Table 6).

**Table 6:** Relation between clinical symptoms and neoplastic status of lesions Values are expressed as frequency (percentage). The association between clinical symptoms and neoplastic status (non-neoplastic vs. pre-neoplastic/neoplastic lesions) was assessed using Fisher’s exact test. A p-value < 0.05 was considered statistically significant. %: Percentage

Clinical features	Non-neoplastic cases; Frequency (%)	Pre-neoplastic/neoplastic cases; frequency (%)	P-value
Abdominal pain	33 (86.84)	5 (13.16)	0.023
Dysphagia	4 (12.90)	27 (87.10)	
Dyspepsia	22 (95.65)	1 (4.35)	
Vomiting	12 (92.30)	1 (7.70)	
Retro-sternal burning	8 (88.89)	1 (11.11)	
Hematemesis	5 (100)	0 (0.0)	
Weight loss	0 (0.0)	6 (100)	
Diarrhea	6 (100)	0 (0.0)	
Loss of appetite	1 (33.33)	2 (66.67)	
Weakness	3 (100)	0 (0.0)	
Oral ulcer	1 (100)	0 (0.0)	

It was found that most of the non-neoplastic lesions belonged to the 41-50 years age group (25.35%). Pre-neoplastic/neoplastic lesions were most frequent in the 51-60 years age group (48.27%). A significant difference was found among age groups (p=0.023). Males were most commonly affected by both non-neoplastic (61.97%) and pre-neoplastic/neoplastic (72.41%) lesions. However, no significant difference was observed between the genders (p = 0.32) (Table 7).

**Table 7:** Relation of age groups and gender with neoplastic status of the upper GI lesions Values are expressed as frequency (percentage). The association between age groups, gender, and neoplastic status (non-neoplastic vs. pre-neoplastic/neoplastic lesions) was analyzed using Fisher’s exact test. A p-value < 0.05 was considered statistically significant. GI: Gastrointestinal; %: Percentage

Parameters	Non-neoplastic cases; Frequency (%)	Pre-neoplastic/neoplastic cases; Frequency (%)	p-value
Age groups (years)			
11–20	3 (4.22)	0 (0.0)	0.023
21–30	12 (16.90)	0 (0.0)	
31–40	14 (19.71)	3 (10.34)	
41–50	18 (25.35)	5 (17.24)	
51–60	16 (22.53)	14 (48.27)	
61–70	6 (8.45)	5 (17.24)	
> 70	2 (2.81)	2 (2.81)	
Gender			
Male	44 (61.97)	21 (72.41)	0.32
Female	27 (38.03)	8 (27.59)	

Twenty-three percent of cases were classified as pre-neoplastic/neoplastic lesions on endoscopic examination, increasing to 29% on histological examination. Similarly, 77% of cases were labeled as non-neoplastic on endoscopy, while 71% were histopathological diagnoses. Accuracy in diagnosing a neoplastic lesion of the upper GI tract by endoscopy was 88%, with substantial agreement with the histopathological diagnosis (Table 8). Sensitivity, specificity, positive predictive value (PPV), and negative predictive value (NPV) for the endoscopic diagnosis of neoplastic lesions were 69%, 96%, 87%, and 88%, respectively (Table 9).

**Table 8:** Concordance of endoscopic diagnosis with histopathological diagnosis (reference standard) for neoplastic lesions of the upper gastrointestinal tract Histopathological diagnosis was used as the reference standard. Kappa statistics were used to assess agreement between endoscopic and histopathological diagnoses. The kappa value was 0.69 (95% CI: 0.52–0.85), indicating substantial agreement, and was statistically significant (p < 0.001). Diagnostic accuracy measures were calculated based on this reference standard.

Diagnostic tools		Histopathological diagnosis		Total
		Pre-neoplastic/neoplastic	Non-neoplastic	
Endoscopic diagnosis	Pre-neoplastic/neoplastic	20	3	23
	Non-neoplastic	9	68	77
Total		29	71	100

**Table 9:** Diagnostic performance of endoscopy for detecting neoplastic lesions using histopathology as the reference standard Diagnostic performance measures were calculated using histopathological diagnosis as the reference standard.

Parameter	Value (%)	95% CI (%)
Sensitivity	69	49.2–84.7
Specificity	96	88.1–99.0
Positive Predictive Value (PPV)	87	66.4–97.2
Negative Predictive Value (NPV)	88	78.7–93.6
Diagnostic Accuracy	88	80.2–93.7

## Discussion

The most common age group for the cases was 51-60 years (60%), followed by 41-50 years (23%). Koirala et al. [7] supported our finding by documenting the highest number of biopsies in the 51-60 and 61-70 year age groups (21.4%), followed by the 41-50 year age group (15.7%). Parikh et al. [8] and Dutta et al. [9] also reported that most patients with upper GI endoscopic biopsies were in the 51-60-year age group. However, in contrast to our finding, Koirala et al. [7] and Goel et al. [10] reported the highest number of cases in the 30-50-year and 21-40-year age groups, respectively. Overall, older age is associated with a higher risk of developing upper GI tract lesions. This finding is consistent with global cancer epidemiological data, which demonstrate an increasing incidence of GI malignancies with advancing age due to cumulative exposure to environmental and lifestyle-related risk factors [11].

Male patients (65%) were the most commonly affected in our study. In comparison, 35% were female patients, with an M:F ratio of 1.86:1. This was consistent with other similar studies documenting a male preponderance, with M:F ratios of 2.1:1 [7], 1.05:1 [8], 2.33:1 [9], and 1.44:1 [10].

The most common clinical presentation of upper GI tract lesions observed was abdominal pain in 38% of the patients, followed by dysphagia (31%), dyspepsia (23%), and vomiting (13%). However, Koirala et al. [7] documented that 72.80% presented with dyspepsia, while 64.20% reported dysphagia. Parikh et al. [8] observed dyspepsia as the most common indication for upper GI endoscopy.

In our study, the most common upper GI tract biopsy site was the stomach (56%), followed by the esophagus (29%), which is comparable to that reported by Koirala et al. [7]. Parikh et al. [8] and Koirala et al. [7] also documented the stomach as the most common site, i.e., 66.66% and 83.76%, respectively, followed by the duodenum, i.e., 22.6% and 10.47%, respectively. However, Goel et al. [10] observed maximum biopsies from the duodenum (36.9%), followed by gastric biopsies (34.9%), in contrast to our finding. In the present study, the most common stomach biopsy site was the antrum (83.9%), followed by the body (8.9%), the fundus (5.4%), and the cardia (1.8%). Koirala et al. [7] also supported our findings, stating that the antrum (68.75%) and body (25.0%) were the most common sites, followed by the fundus (3.75%) and pylorus (2.5%).

Ulcer was the most common upper GI endoscopic finding (26%), followed by erosion (14%), in our study. A previous study [12] observed growth in 40% of the cases on endoscopic examination, followed by erythema (36%). We observed that among 29 esophageal endoscopic examinations, proliferative and ulcero-proliferative growth were the most common endoscopic findings (each 27.5%). Koirala et al. [7] mostly observed multiple erosions (50%) on esophageal endoscopy. In our study, among 56 endoscopic examinations of the stomach, ulcer was the most common endoscopic finding, i.e., in 30.4% of patients. Koirala et al. [7], however, showed mostly erythema (34.37%) on gastric endoscopy. We observed that ulcer was the most common finding in 14 duodenal endoscopic examinations (42.9%), followed by flattening of the mucosa (21.5%). Koirala et al. [7] noted erosion to be the most common (55%) duodenal endoscopic finding, followed by ulcerative mucosa (15%). Only one patient underwent endoscopic biopsy of the GE junction, and erythema was observed in that case. Koirala et al. [7] also had only one GE junction biopsy, but with an erosive lesion.

Overall, 77% of cases were clinically suspected to be non-neoplastic lesions, and 23% were neoplastic; 58.60% of the esophageal lesions, 7.10% of the stomach lesions, and 14.3% of the duodenal lesions were clinically suspected to be neoplastic, and the rest were non-neoplastic lesions. The GE junctional lesion was clinically non-neoplastic. Overall, Goel et al. [10] suspected 71.60% non-neoplastic lesions and 28.40% neoplastic lesions clinically. They clinically suspected that 57.33% of esophageal lesions, 19.35% of stomach lesions, and 12.24% of duodenal lesions were neoplastic. On histopathology, 71% were diagnosed as non-neoplastic lesions (most common), 4% as pre-neoplastic lesions, and 25% as neoplastic lesions. Koirala et al. [7] observed 48.57% non-neoplastic lesions, 7.20% pre-neoplastic lesions, and 5.20% neoplastic lesions under the microscope. Parikh et al. [8] diagnosed 94.11% as non-neoplastic (most common) and only 5.89% as neoplastic lesions on histology. In contrast to our findings, Dutta et al. [9] confirmed that neoplasia was the most common histological finding (48.33%), followed by non-neoplasia (41.67%) and pre-neoplasia (5%).

Overall, in the present study, no specific pathology was found under the microscope (32%). Chronic gastritis was the most common (13%) diagnosis. The most common non-neoplastic lesions were diagnosed with non-specific pathology, followed by chronic gastritis. Moderate dysplasia and moderately differentiated SCC were the most common pre-neoplastic and neoplastic lesions, respectively. Dutta et al. [9] also found gastritis, dysplasia, and SCC to be the most common non-neoplastic, pre-neoplastic, and neoplastic diagnoses on histology, respectively. Koirala et al. [7] found that most commonly, chronic gastritis (non-neoplastic), Barrett’s esophagus (pre-neoplastic), and adenocarcinoma of the stomach (neoplastic) were found on histology. The relatively high proportion of non-specific pathology observed in the present study may partly reflect sampling limitations, superficial biopsy specimens, or incomplete clinicopathological correlation in certain cases.

In the present study, on the histology of esophageal biopsies, we observed 20.7% non-neoplastic, 13.8% pre-neoplastic, and 65.5% neoplastic pathology, which is consistent with that reported by Devendrappa et al. [12]. Overall, the most common esophageal histopathological diagnosis was moderately differentiated SCC (27.5%). The most common non-neoplastic lesion was acute esophagitis, supported by Koirala et al. [7]. However, Devendrappa et al. [12] documented chronic esophagitis, and Parikh et al. [8] showed that reflex esophagitis is the most common non-neoplastic histologic lesion. Moderate dysplasia was the most commonly diagnosed esophageal pre-neoplastic lesion in our study; however, Koirala et al. [7] reported low-grade dysplasia (pre-neoplastic) as the most common. Moderately differentiated SCC was the most common esophageal neoplastic lesion diagnosed by us, comparable to other studies [7-9,12].

In a histological analysis of 56 gastric biopsies, we observed 89.3% non-neoplastic and 10.7% neoplastic pathology, similar to Devendrappa et al. [12]. Overall, the most common gastric histopathological diagnosis, as well as the non-neoplastic diagnosis, was no specific pathology (44.6%), followed by chronic gastritis (23.2%) and neoplastic adenocarcinoma NOS. Our findings were comparable to those of previous studies [7-10]. We found mucinous adenocarcinoma as the second most common neoplastic lesion; however, Parikh et al. [8] and Koirala et al. [7] reported signet ring cell adenocarcinoma as the second most common neoplastic lesion in their patients. Devendrappa et al. [12] found poorly differentiated adenocarcinoma to be the most common in the stomach. Only one GE junctional biopsy was performed, which showed non-specific histopathology, similar to that reported by Koirala et al. [7].

On histological diagnosis of 14 duodenal biopsies, we found all lesions to be non-neoplastic (100%), with chronic duodenitis the most common type (35.7%), in agreement with previous studies [7,8,10-12]. However, in contrast, Dutta et al. [9] found that duodenal tuberculosis was most commonly diagnosed histopathologically.

We also screened all cases for *H. pylori*. Routine H&E stains may miss *H. pylori*, so special stains should be used for its detection. Therefore, the use of Giemsa stain should be considered to visualize *H. pylori* better. No *H. pylori* cases were reported on routine H&E. Consequently, Giemsa stain was used, and overall 7% (7/100) of cases were *H. pylori* positive. 46% of the total chronic gastritis cases and 20% of the total chronic superficial gastritis cases were *H. pylori* positive. Overall, 30.43% of all cases of gastritis were *H. pylori* positive. Parikh et al. [8] and Koirala et al. [7] reported 5.6% and 46.21% of total cases of chronic gastritis as *H. pylori*-positive, respectively, while Goel et al. [10] found that 14.2% and 35.3% of cases of gastritis were *H. pylori*-positive, respectively. The low detection rate of this infection in our study could be due to better hygiene and sanitation, better food handling and storage, high awareness and early detection, widespread use of antibiotics and proton pump inhibitors, and empirical treatment with these agents before undergoing endoscopic biopsy. These medications can suppress or partially eliminate *H. pylori*, leading to false-negative results on biopsy. Additionally, reliance solely on histopathological examination with Giemsa staining without adjunctive diagnostic methods may have underestimated the true prevalence of *H. pylori* infection. The heterogeneity in *H. pylori* prevalence across studies could be due to factors such as geography, age, ethnicity, and population. Global meta-analyses have also reported significant regional variation in *H. pylori* prevalence, influenced by socioeconomic status, hygiene practices, and healthcare access [13].

Most of the non-neoplastic and pre-neoplastic/neoplastic lesions were observed in the 41-50 years (25.35%) and 51-60 years (48.27%) age groups. A significant difference was found among age groups (p=0.023). Other similar studies also documented the predominance of neoplastic lesions in the higher age group [7-9]. We have not observed any significant difference between the genders for the incidence of neoplastic lesions. Dysphagia and weight loss were most commonly associated with pre-neoplastic/neoplastic lesions, as supported by Parikh et al. [8].

Accuracy for diagnosing a neoplastic lesion of the upper GI tract by endoscopy was 88%, with substantial agreement with the histopathological diagnosis (concordance = 68%). Nine lesions, clinically and endoscopically classified as non-neoplastic, were neoplastic on histology, while three neoplastic lesions were labeled as non-neoplastic under the microscope. The sensitivity, specificity, PPV, and NPV for the endoscopic diagnosis of neoplastic lesions were 69%, 96%, 87%, and 88%, respectively. Our findings are comparable to those of Goel et al. [10]. Similar studies have also demonstrated a strong correlation between endoscopic findings and histopathological diagnosis, highlighting the complementary role of these modalities in the evaluation of upper GI lesions [14].

This study had several limitations. The small sample size and short study duration may not fully represent the population, limiting the generalizability of the findings. Additionally, it was a single-center study, which may limit the generalizability of the results to other regions with different populations. The lack of long-term follow-up prevented evaluation of the prognosis and progression of lesions after diagnosis. Furthermore, there may have been an underestimation of *H. pylori* infection, as factors such as prior antibiotic or proton pump inhibitor use, biopsy site and technique, and reliance solely on histological staining could have reduced detection rates. More sensitive tests, such as the rapid urease test, the urea breath test, or PCR, were not used. Information regarding prior proton pump inhibitor or antibiotic therapy before endoscopy was not consistently available and may have influenced the detection rate of Helicobacter pylori. The absence of a standardized gastric biopsy protocol may have contributed to sampling variability and reduced detection of focal lesions or *H. pylori* colonization.

## Conclusions

This study highlights the pivotal role of upper GI endoscopic biopsy in the evaluation of mucosal lesions, enabling early detection of premalignant and malignant conditions while effectively differentiating them from non-neoplastic lesions. Although endoscopy serves as an essential initial diagnostic modality for visualization and lesion localization, histopathological examination remains the gold standard for definitive diagnosis. The integration of endoscopic findings with histopathology significantly enhances diagnostic accuracy and guides appropriate clinical management. Furthermore, factors such as increasing age and clinical presentations like dysphagia were found to be strongly associated with neoplastic pathology. Future multicentric studies with larger sample sizes and long-term follow-up are warranted to validate these findings and improve early detection strategies in high-risk populations. However, the findings should be interpreted cautiously due to the relatively small sample size and the single-center design of the study.
